# Identification of Chalcones as *Fasciola hepatica* Cathepsin L Inhibitors Using a Comprehensive Experimental and Computational Approach

**DOI:** 10.1371/journal.pntd.0004834

**Published:** 2016-07-27

**Authors:** Florencia Ferraro, Alicia Merlino, Nicolás dell´Oca, Jorge Gil, José F. Tort, Mercedes Gonzalez, Hugo Cerecetto, Mauricio Cabrera, Ileana Corvo

**Affiliations:** 1 Laboratorio de Investigación y Desarrollo de Moléculas Bioactivas, Departamento de Ciencias Biológicas, CENUR Litoral Norte, Universidad de la República, Paysandú, Uruguay; 2 Laboratorio de Química Teórica y Computacional, Instituto de Química Biológica, Facultad de Ciencias, Universidad de la República, Montevideo, Uruguay; 3 Departamento de Genética, Facultad de Medicina, Universidad de la República, Montevideo, Uruguay; 4 Laboratorio de Reproducción Animal, Producción y Reproducción de Rumiantes, Departamento de Ciencias Biológicas, CENUR Litoral Norte-Facultad de Veterinaria, Universidad de la República, Paysandú, Uruguay; 5 Grupo de Química Medicinal, Laboratorio de Química Orgánica, Facultad de Ciencias, Universidad de la República, Montevideo, Uruguay; 6 Área de Radiofarmacia, Centro de Investigaciones Nucleares, Facultad de Ciencias, Universidad de la República, Montevideo, Uruguay; McGill University, CANADA

## Abstract

**Background:**

Increased reports of human infections have led fasciolosis, a widespread disease of cattle and sheep caused by the liver flukes *Fasciola hepatica* and *Fasciola gigantica*, to be considered an emerging zoonotic disease. Chemotherapy is the main control measure available, and triclabendazole is the preferred drug since is effective against both juvenile and mature parasites. However, resistance to triclabendazole has been reported in several countries urging the search of new chemical entities and target molecules to control fluke infections.

**Methodology/Principle Findings:**

We searched a library of forty flavonoid derivatives for inhibitors of key stage specific *Fasciola hepatica* cysteine proteases (*Fh*CL3 and *Fh*CL1). Chalcones substituted with phenyl and naphtyl groups emerged as good cathepsin L inhibitors, interacting more frequently with two putative binding sites within the active site cleft of the enzymes. One of the compounds, **C34**, tightly bounds to juvenile specific *Fh*CL3 with an IC_50_ of 5.6 μM. We demonstrated that **C34** is a slow-reversible inhibitor that interacts with the Cys-His catalytic dyad and key S_2_ and S_3_ pocket residues, determinants of the substrate specificity of this family of cysteine proteases. Interestingly, **C34** induces a reduction in NEJ ability to migrate through the gut wall and a loss of motility phenotype that leads to NEJ death within a week *in vitro*, while it is not cytotoxic to bovine cells.

**Conclusions/Significance:**

Up to date there are no reports of *in vitro* screening for non-peptidic inhibitors of *Fasciola hepatica* cathepsins, while in general these are considered as the best strategy for *in vivo* inhibition. We have identified chalcones as novel inhibitors of the two main Cathepsins secreted by juvenile and adult liver flukes. Interestingly, one compound (**C34)** is highly active towards the juvenile enzyme reducing larval ability to penetrate the gut wall and decreasing NEJ´s viability *in vitro*. These findings open new avenues for the development of novel agents to control fluke infection and possibly other helminthic diseases.

## Author Summary

Despite the widespread prevalence of liver fluke infections and many efforts aimed at developing a preventive vaccine just one drug, triclabendazole, is effective in killing all parasite stages. This is aggravated by the increasing number of countries reporting parasite isolates resistant to triclabendazole treatment, highlighting the urgent need to develop new agents to control fluke infections. Here, we report the discovery of chalcones that effectively inhibits key cysteine proteases essential for parasite development and survival within the host. We further characterized the mode of inhibition of the most active compound against cathepsins secreted by both adult and larval stages of the parasite. This class of compounds is generally considered safe for clinical use and we showed that the most effective derivative is not cytotoxic to bovine sperm cells. Importantly, *in vitro* approaches showed that it reduces host penetration and larvae viability, finally leading to parasite death. These findings point at this derivative as a good starting point for the development of novel agents to control fluke infection and possibly other helminthic diseases.

## Introduction

Parasitic flatworms are the causative agents of serious human and livestock infections many of which have been considered neglected tropical diseases in urgent need for research efforts. Liver flukes (*Fasciola spp*.) cause fasciolosis, traditionally a relevant disease of cattle, sheep and goats [1], currently considered an emerging zoonotic disease by the World Health Organization due to an increased incidence of human infections [2]. Despite many efforts to develop a vaccine to prevent mammalian infection [3], chemotherapy is the only *Fasciola* control mechanism currently available. Triclabendazole is the first choice drug since it is effective in killing juvenile and mature parasites, but resistance is emerging in several countries [4, 5]. This highlights the urgency of finding novel strategies and target molecules for developing innovative drugs to treat fluke infections.

Many virulence factors have been identified as primary targets for parasite control, since they can be used for developing therapies based on drugs or immunogens. Cysteine proteases play essential roles in numerous protozoan (like *Trypanosoma cruzi* and *Plasmodium falciparum*) and other helminth parasites [6], were they have been explored as appropriate targets for antiparasitic chemotherapy [7, 8, 9, 10, 11]. Liver flukes secrete high amounts of cysteine proteases being key players in host-parasite interaction at all stages of their life cycle [12, 13, 14, 15]. Cathepsin L3 is expressed by the newly excysted juveniles (NEJ) and consequently take part in the first steps of mammalian host infection [12, 13, 15, 16]. A different set of cathepsins L are produced by adult flukes residing in the bile ducts [15,16] and are involved mainly in digesting nutrients and developing immune responses mechanisms [17, 18]. Therefore, *F*. *hepatica* cathepsins are interesting targets for drug development in an effort to avoid parasite infection or reduce parasite burden and the pathogenic effects of the infection.

Due to their role in human disease and tumour progression, inhibitors targeting cysteine proteases have been extensively studied. Most efforts were focused on peptidic inhibitors with different substituents such as aminoacetonitriles, heterocyclic ketones, nitriles, epoxides and vinyl sulfones [19, 20, 21]. Many of these small molecules contain electrophilic groups that bind in the active-site through covalent interactions with the catalytic cysteine either in a reversible or irreversible way. Non-peptidic compounds have also been reported as cathepsin inhibitors, which are considered a better strategy for *in vivo* inhibition in order to avoid degradation by proteases. Among these, chalcones and other flavonoids can be found [22, 23, 24]. Flavonoids are biologically active compounds that possess remarkable properties, being presented as antioxidant, anticancer, antidiabetes, anti-inflammatory, antiprotozoal, antiHIV, antituberculosis, among many other interesting activities [25, 26, 27]. What is more, several flavonoids, particularly chalcones, have shown good pharmacological potential and have been approved for clinical use or tested in humans [27]. There have also been described flavonoid derivatives with cathepsin L-like cysteine protease inhibitory activity [23, 28, 29, 30, 31] as well as some natural flavonoids with fasciolicide activity [32, 33]. However, up to date there are no reports of *in vitro* screening for non-peptidic inhibitors of *Fasciola* cathepsins (*Fh*CLs) or *in vitro* screening of synthetic chalcones with fasciolicide activity.

Taking this into account, we performed a search for small molecular weight compounds from our own library of synthetic flavonoids that may inhibit key *Fasciola* cysteine proteases as *Fh*CL3 and *Fh*CL1. *Fh*CL3 is the only cathepsin L found in the early NEJ excretion/secretion products [12, 13, 14, 16], and *Fh*CL1 is the main cathepsin expressed by adult flukes [14, 15]. This kind of compounds was also previously assayed as anti-tumoral and cancer chemopreventive agents showing non-mutagenic effects and being well tolerated *in vivo* [34, 35, 36]. Here, we identified novel inhibitors of *F*. *hepatica* cathepsins with *in vitro* fasciolicide activity which shall contribute in the design of novel drugs to control fluke infection.

## Methods

### Selection of compounds

Since flavonoids have been reported as able to inhibit cysteine protease family enzymes, we evaluated 39 synthetic flavonoids (S1–S3 Tables) from our chemical library. In order to test a variety of chemical entities, we included chalcones without (**C1-C8**, **C20** and **C21**) or with (**C9-C19** and **C22-C26**) a 2'-substituent in the A ring, chalcones with extended aromaticity (**C27-C35**) and flavones (**C36-C39**) along with the natural flavonol quercetin (**C40**).

### Production of recombinant *Fh*CL1 and *Fh*CL3

*Fh*CL1 and *Fh*CL3 recombinant enzymes were expressed in the yeast *Hansenula polymorpha* as previously described [17, 39]. Briefly, yeast transformants were cultured in 500 mL YEPD broth (1% glucose, 1% tryptone, 1% yeast extract) at 37°C to an OD_600_ of 2–6, harvested by centrifugation at 3000xg for 10 min and induced by resuspending in 50 mL of buffered minimal media (0.67% yeast nitrogen base; 0.1M phosphate buffer pH 6.0; 1% methanol) for 36 h at 30°C. Recombinant propeptidases were secreted to the culture media, and recovered by 20–30 fold concentration of culture supernatants by ultrafiltration with a 10 kDa cut-off membrane. The proenzymes were autocatalytically activated to the mature form by incubation for 2 h at 37°C in 0.1 M sodium citrate buffer (pH 5.0) with 2 mM DTT and 2.5 mM EDTA, dialyzed against PBS pH 7.3 and stored in aliquots at -20°C until used. Functionally active recombinant enzyme was determined by titration against the cysteine protease inhibitor E-64c.

### *Fh*CL1 and *Fh*CL3 inhibition screening of the selected compounds

A 10 mM stock of each evaluated compound was prepared in dimethylsulfoxide (DMSO). Enzyme activity assays were conducted to evaluate the inhibitory capacity of the different compounds. *Fh*CL1 and *Fh*CL3 were used at nanomolar concentrations to measure initial rates during the first 10 min of the assay and compounds were tested at a moderate fixed dose of 10 μM. Briefly, each enzyme and compound were preincubated 5 minutes in a 96-well plate in 0.1M sodium phosphate buffer pH 6, 1 mM DTT and 1 mM EDTA at room temperature. The reaction was initiated by the addition of 20 μM of substrate, a synthetic peptide conjugated to the fluorophore 7-amino-4-methylcoumarin (AMC). Enzyme activity was measured by the increase in AMC fluorescence as peptide substrates were hydrolyzed (Z-VLK-AMC for *Fh*CL1 and Tos-GPR-AMC for *Fh*CL3) at an excitation wavelength of 340 nm and emission wavelength of 440 nm using a spectrofluorometer (Varioskan Thermo). Enzyme activity was expressed as RFU/s (relative fluorescence units of AMC released per unit of time). Each compound was tested in duplicate. A progress curve without enzyme was performed to control for non-catalyzed reactions between substrates and inhibitors and a spectrum from 300 to 450 nm wavelength was done for each inhibitor to verify that none of the compounds has optical activity in the measurement range. The percentage of enzyme inhibition was calculated as: 100 - (v_i_/v_o_) x 100, where v_i_ and v_o_ correspond to the initial rate of AMC fluorescence increase (RFU/s) with and without inhibitor, respectively.

### Further C34 inhibition studies

IC_50_ was calculated at 12 different concentrations of the inhibitor compound, 0, 0.625, 0.937, 1.25, 1.875, 2.5, 3.75, 5, 7.5, 10, 15 and 20 μM. The measurement of enzyme activity was performed in triplicate in a 96-well plate as previously described. We plotted initial rates (RFU/s) versus log_10_ of inhibitor concentration and obtained the IC_50_ value from a linear regression of the data. We also carried out slow-binding assays to evaluate if inhibition was time-dependent. **C34** at 10 μM was incubated with each enzyme for increasing lengths of time from 3 to 120 min and the percentage of inhibition was determined as described for the screening assay. To test for reversibility of the enzyme-compound interaction, we performed rapid dilution assays [37]. Briefly, samples containing each enzyme at a 100-fold concentration (compared with standard assays) were preincubated with 10-fold the IC_50_-equivalent concentration of the inhibitor for 20 min at room temperature. Control reactions for each enzyme without inhibitor were carried out in parallel. Samples were then diluted 100-fold with assay buffer containing the appropriate substrate to initiate reactions, and the time course of AMC release was measured as previously describe.

### Preparation of protein structures for molecular docking

*Fh*CL1 and *Fh*CL3 structures previously obtained by homology modelling were used [38, 39] (Robinson 2011, Corvo 2013). In order to improve structures accuracy for molecular docking, MD simulations were performed using the *pmemd* module implemented in the AMBER12 package [40], with the *ff03*.*r1* force field [41]. Hydrogen atoms and sodium ions (to neutralize charge) were added to each protein with the *leap* utility. Each system was placed in a truncated octahedral box of TIP3P explicit water [42], extended 10 Å outside the protein on all sides. The structures of *Fh*CL1 and *Fh*CL3 were treated as follows: a) water and counterions were relaxed to minimize energy during 2,500 steps (500 steepest descent steps, SD, and 2,000 conjugate-gradient steps, CG) with the protein restrained with a force constant of 500 kcal/molÅ^2^; b) the system was minimized without restraints during 20,000 steps (5,000 SD and 15,000 CG). Long range electrostatic interactions were considered using the particle-mesh Ewald (PME) method [43] and a non-bonded interactions cutoff of 10 Å was used. After minimization, each system was gradually heated in a NVT ensemble from 0 to 300 K over 100 ps using the Berendsen coupling algorithm [44]. This procedure was followed by 20 ns of NPT simulations at 300 K and 1 atm pressure using the Langevin dynamics algorithm [45]. All bonds involving hydrogen atoms were constrained using the SHAKE algorithm [46]. The equations of motion were integrated with a time step of 2.0 fs and coordinates of the systems were saved every 2 ps. Representative structures of *Fh*CL1 and *Fh*CL3 from the last 30 ns of the trajectories were obtained through cluster analysis using the average-linkage algorithm [47] and used for subsequent docking calculations. Clustering, RMSD, RMSF and hydrogen bond analysis were performed using the *cpptraj* module in AmberTools14. For trajectories visualization the VMD program was used [48].

### Preparation of ligand structures

Compounds **1**–**39** (S1–S3 Tables) were fully optimized at the ωB97XD/6-31+G(d,p) level [49, 50] in water using the IEF-PCM continuum model [51] with Bondi atomic radii. The nature of the optimized structures as stable species was inspected checking the *eigenvalues* of the analytic Hessian matrix, calculated at the same level of theory, to be positive in all the cases. All these calculations were performed using the Gaussian09 software [52].

### Ligand-protein molecular docking

To predict the binding site of flavonoids **C1**-**C39** into *Fh*CL1 and *Fh*CL3 flexible-ligand docking was performed using a grid box of 126×94×116 points with a grid spacing of 0.60 Å in order to cover the entire protein surface (blind docking). The grid box was centered on the macromolecule. Results differing by less than 2.0 Å in root-square deviation were grouped in the same cluster. The conformation with the lowest binding energy was chosen from the most populated cluster and the corresponding ligand-protein complex was used for further MD studies. All docking calculations were done with the AutoDock 4.2 [53] software package using the Lamarckian genetic algorithm. A population size of 150 individuals and 2.5×10^6^ energy evaluations were used for 50 search runs.

### Ligand-protein molecular dynamics

MD simulations of **C34** with *Fh*CL1 and *Fh*CL3 were performed as described above using the GAFF [54] force field for the ligand. RESP partial charges [55] for **C34** were derived using Gaussian09 at the HF/6-31G* level and the *antechamber* module in AMBER12 was employed to obtain the force field parameters. 40 ns of productive MD were simulated and coordinates of the systems were saved every 10 ps.

### *In vitro* NEJ treatment with C34 and gut invasion assay

*F*. *hepatica* metacercariae were acquired from Baldwin Aquatics Inc. (Monmouth, Oregon) for *in vitro* NEJ treatment and from Instituto Miguel C. Rubino (DILAVE, MGAP, Uruguay) for the gut invasion assay. NEJ were obtained by *in vitro* excystement as previously described with minor modifications [56], with no differences observed between metacercariae from different origin. Briefly, 100 metacercariae were incubated with 1% sodium hypochlorite for 5 min at room temperature to remove the outer cyst wall and then washed exhaustively with PBS. Metacercarie were activated by incubation at 39°C in a medium prepared by mixing equal volumes of solution A (0.4% sodium taurocholate, 120 mM NaHCO_3_, 140 mM NaCl pH 8.0) and solution B (50 mM HCl and 33 mM L-cysteine). A 100 μm filters were used to retain the cyst wall as NEJ began to emerge. The excystment process was monitored for m90-180 min under the microscope. Collected NEJ were washed three times with RPMI-1640 supplemented with 200 U/mL Penicillin G sulfate, 200 mg/mL streptomycin sulfate, 500 ng/mL amphotericin B, 10 mM HEPES, counted and divided in groups of around 20 parasites that were transferred to 12 wells tissue culture plates. Parasites were maintained at 37°C, 5% CO_2_ in modified Basch’s medium [57]. At day 1, **C34** (50 μM) was added to treated groups and 0.5% DMSO to control groups, each condition tested by duplicate. Medium was changed twice a week and fresh compound was added, control NEJ were cultured for 20 days. NEJ behavior was monitored under a light microscope (Olympus BX41), every day each well was recorded for a minute in order to assess parasite motility and registered using the following score: 3-normally active; 2- reduced activity (sporadic movement); 1- immotile (adapted from [58]). For the gut invasion assay we performed an *in vitro* excystement of metacercarie as previously described and incubated the NEJ for 4 h either in the presence of 50 μM **C34** or 0.5% DMSO (control group) and immediately transferred them to gut sacs. For gut preparation we used the protocol previously described by Burden *et al*. with some modifications [59]. Brifely, 6 weeks old male Wistar rats were euthanized by cervical dislocation and approximately 25 cm of small intestine was excised using the caecum as a reference. The intestine was washed several times with RPMI-1640 media warmed at 37°C and cut in 5 cm sacs. One end of each sac was ligated, 20–30 NEJ from treated or control groups were pipetted inside the sac and then the other end was ligated. Gut sacs were maintained in RPMI-1640 media at 37°C, 5% CO_2_ in 12 wells plates and parasites that migrated through the mucosal wall within 3 h were recovered at the plate bottom and counted. Each condition was assayed in triplicate.

### Cytotoxicity assay on bovine spermatozoa

Semen samples were obtained from a healthy fertile Hereford bull and kept frozen in 0.5 mL straws (extended in Andromed, Minitube, Germany) under liquid nitrogen until use. The semen used belonged to a single freezing batch that was obtained during a regular collection schedule with an artificial vagina. Samples from three straws were thawed and a sperm pool was prepared in PBS at a concentration of 40 million spermatozoa per mL, then 50 μL of this sperm suspension was carefully mixed with 50 μL of **C34** diluted to 100, 50, 25, 12.5 and 6.25 μM or with 1% DMSO in control experiments. Each condition was assayed by duplicate in 96-well plates and controls were assayed by triplicate. Plates were incubated at 37°C for 1 h with moderate shaking. The motility analysis was carried out using a CASA (Computer Assisted Semen Analyzer) system Androvision (Minitube, Tiefenbach, Germany) with an Olympus BX 41 microscope (Olympus, Japan) equipped with a warm-stage at 37°C. Each sample (10 μL) was placed onto a Makler Counting Chamber (deph 10 μm, Sefi-Medical Instruments, Israel) and the following parameters were evaluated: percentage of total motile spermatozoa (motility >5 μm/s) and velocity curved line (VCL, >24 μm/s). At least 400 spermatozoa were analysed from each sample from at least four microscope fields.

## Results and Discussion

### Aromatic chalcones are good cathepsin inhibitors

**Fig 1:**
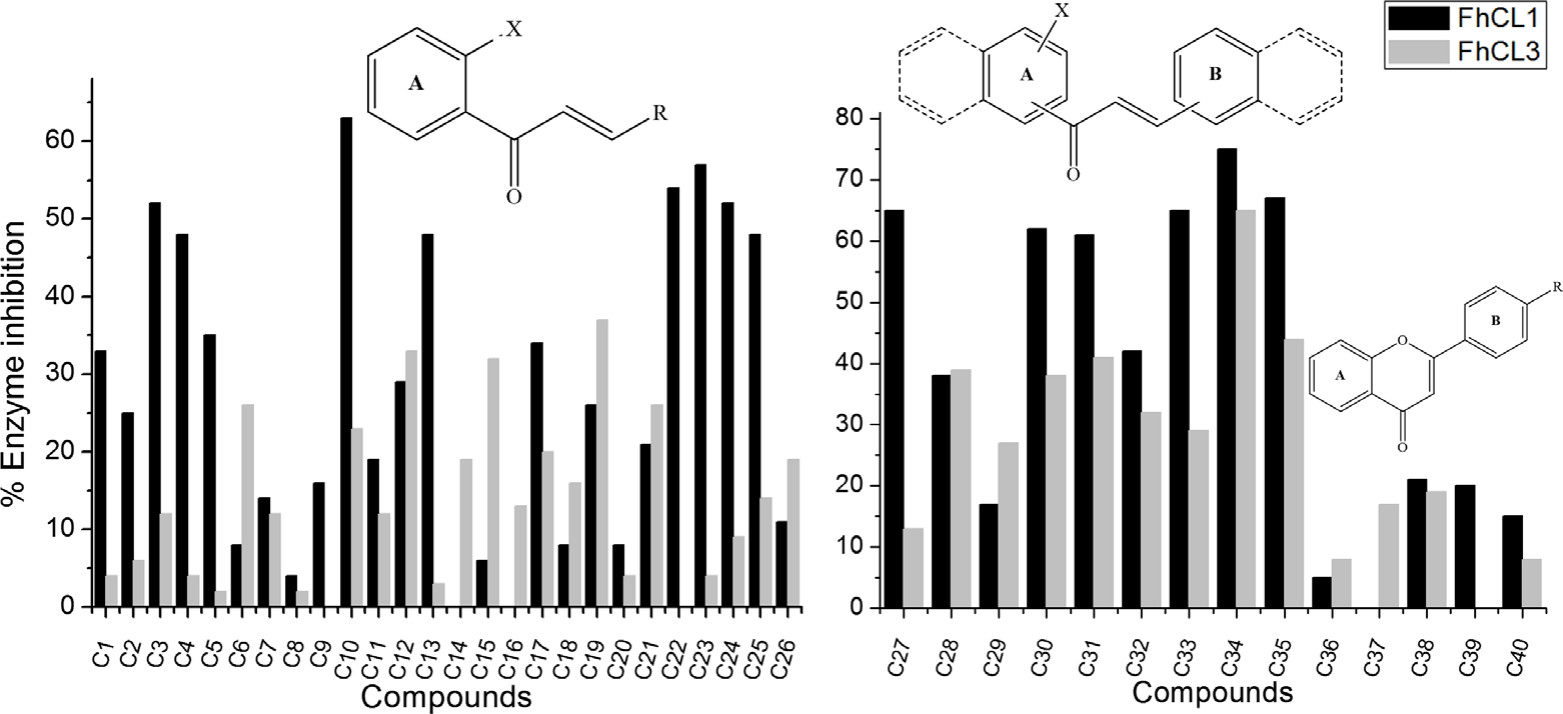
Screening of chalcones as *Fh*CL1 and *Fh*CL3 inhibitors. The % of inhibition of *Fh*CL1 (black bars) and *Fh*CL3 (gray bars) activity is shown for each compound of: **A.** cluster I (chalcones modified on ring B, represented as R), **B.** clusters II and III (naphtochalcones and flavone derivatives, respectively). All compounds were screened at 10 μM. A general structure of each cluster of compounds is shown above the graphic bars: **A.** X can be -H or -OH and R is phenyl with different substituents or a heterocycle. **B.** X can be -H or -OH at position 1 or 2, dashed lines represent phenyl or naphtyl at position 1 or 2 and R is -H, -Cl, -Br or -OCH_3_. **C40** is the natural flavone quercetine (the structure of each compound can be found in S1–S3 Tables).

We evaluated forty flavonoids from three structural clusters of our own chemistry library as inhibitors of *Fh*CL1 and *Fh*CL3. Cluster **I** contained twenty-six chalcones modified in ring B (**C1**-**C26**, Fig 1A), cluster **II** contained naphtolchalcone derivatives (**C27**-**C35**, Fig 1B) and cluster **III** was composed of flavones modified in ring B (**C36**-**C40**, Fig 1B). Initially, we screened the compounds at a low concentration of 10 μM measuring initial rates during the first 10 min, finding activities ranging from 0% to 75% enzyme inhibition (Fig 1). Roughly, a larger amount of compounds decreased *Fh*CL1 activity considerably, while *Fh*CL3 proved more difficult to inhibit. Thus, eleven compounds showed more than 50% inhibition of *Fh*CL1 (**C3**, C**10**, **C22-C24**, **C27**, C**30, C31, C33, C34** and **C35**) (Fig 1), while two of them, belonging to cluster **II**, were above this cut-off for *Fh*CL3, **C34** and **C35** (Fig 1B), being **C34** the best global inhibitor. None of the assayed flavones (cluster **III**) displayed relevant activities against both *Fh*CL1 and *Fh*CL3 (% inhibition at 10 μM lower than 20%, Fig 1B) for this reason the studied population of flavones was lower than the chalcones one.

In general, chalcones containing heterocycles as B-ring in cluster **I** (**C22-C26**) and those with extended aromaticity belonging to cluster **II** showed the highest inhibition percentages. Clearly, this kind of compounds inhibited more readily *Fh*CL1 than *Fh*CL3 (fourteen showing at least 50% inhibition of *Fh*CL1 *vs* two with *Fh*CL3 at 10 μM). These might be explained considering the active site pocket structure and substrate preferences of the enzymes. While *Fh*CL1 has a wide S_2_ pocket which easily accommodates bulky and aromatic residues, *Fh*CL3 has a narrow and restricted site that preferentially interacts with small moieties like proline and glycine [39]. Strikingly, **C34** substituted by a hydroxyl at position 2 of the A-ring and with a naphthyl on both rings, was the best inhibitor of *Fh*CL3, showing 75% inhibition for *Fh*CL1 and 65% for *Fh*CL3 in the screening. Likewise, the highest percentages of *Fh*CL3 inhibition correspond to the same type of compounds (**C28**, **C30**, **C31**, **C35**) (Fig 1B). We hypothesized that the narrower conformation of *Fh*CL3 active site compared to *Fh*CL1 diminishes the number of compounds that manage to accommodate in such a position as to allow enzyme inhibition.

There are some interesting compounds showing selectivity towards *Fh*CL1, for example derivatives **C4**, **C13** and **C22-C25** exhibiting around 50% inhibition of the former enzyme and poor or no inhibition of *Fh*CL3. These compounds belong to the group containing heterocycles as B-ring (**C22-C25**) or 4-methoxy moiety on the B-phenyl ring (**C4**, **C13**), that might establish hydrogen bonds with specific residues of this enzyme (S4 Table). Particularly, **C20**, the non-hydroxylated analogue of **C22**, does not inhibit *Fh*CL1, suggesting the 2´-OH might also be playing an important role in this inhibition. A chalcone with extended aromaticity (**C27**) also exhibits 65% inhibition of *Fh*CL1 and only 13% of *Fh*CL3, being the only one with a phenyl substituent as B-ring. Notably, among these bulky compounds, the less active towards *Fh*CL1 was **C29**, which lacks Michael-acceptor motive susceptible of nucleophilic attack by enzyme residues (Fig 1B, S2 Table).

The *p*-Cl substituted chalcone **C10** also showed good inhibition for *Fh*CL1 (63%) (Fig 1A, S1 Table). Likewise, this compound offers some interesting structure-activity relationships. Here, a positive influence of the 2´-OH in the inhibition might be addressed again when comparing **C10** with its de-2´OH analogue **C2** that exhibits only 25% of inhibition. Besides, its bioisostere *p*-Br substituted on the B-ring (**C11**) depicts poor inhibition of *Fh*CL1 (19%), suggesting that it is the combination of both 2´-OH and *p*-Cl substituents that exerts optimum inhibition of this enzyme (Fig 1A, S1 Table).

### Different mode of inhibition of C34 with *Fh*CL1 and *Fh*CL3

We selected **C34** to perform additional characterization as it was the best inhibitor of both target enzymes. Consequently, a dose-response study was performed. The IC_50_ was similar with both enzymes, being 7.7 μM and 5.6 μM for *Fh*CL1 and *Fh*CL3 respectively (Table 1). Interestingly, the interaction mode with each enzyme seems to differ. Longer incubation times at 10 μM resulted in higher inhibition of *Fh*CL3 but not *Fh*CL1, thus **C34** interacts in a slow-binding time-dependent fashion only with the former enzyme. Furthermore, when increasing the preincubation time with the enzyme, *Fh*CL3 is almost completely inhibited (96%) (Table 1).

**Table 1:** IC_50_ and effect of preincubation time, at 10 μM, on enzyme inhibition by C34.

		% inhibition over time (min)				
Enzyme	IC_50_ (μM)	3	30	60	90	120
*Fh*CL1	7.7 ± 0.4	73	81	75	67	79
*Fh*CL3	5.6 ± 0.1	53	87	96	96	93

**Fig 2:**
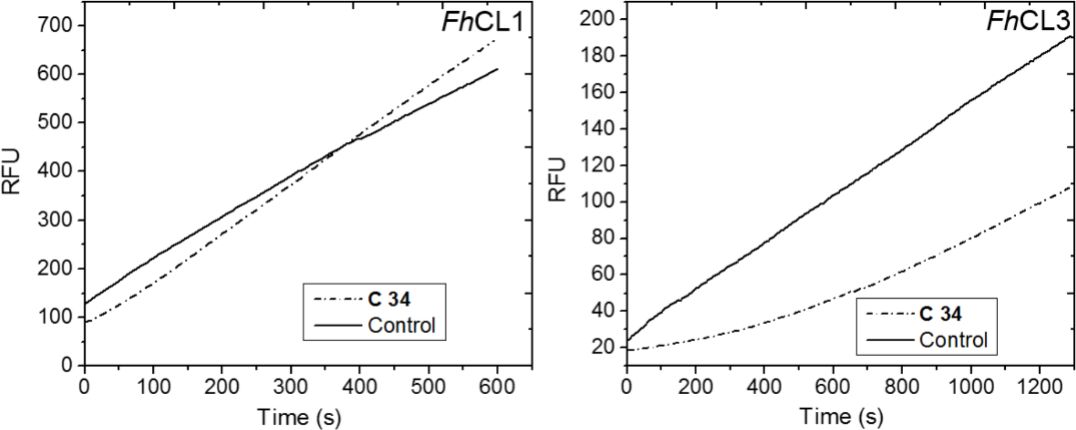
Reversibility of C34 inhibition. Rapid dilution assay to test reversibility of **C34** interaction with cathepsins. Recovery of enzymatic activity was measured after incubation of each enzyme in the absence (Control) or presence of inhibitor (**C34**). Initial rates were measured for 10–20 min after dilution of the enzyme-inhibitor complex with substrate to initiate reaction, and plotted as Relative Fluorescence Units (RFU) of AMC released from substrate per unit of time (s).

We then evaluated the reversibility of the inhibition by measuring the recovery of enzymatic activity after a rapid and large dilution of the enzyme–inhibitor complex. When the initial rate of the reaction is similar in the presence and absence of the inhibitor, it indicates a rapid recovery of activity after compound dilution meaning the reaction is reversible. This was the case for the interaction of **C34** with *Fh*CL1 (Fig 2, left panel), where the progress curve after inhibitor dilution has a slope similar to that of the control sample (enzyme incubated and diluted in the absence of inhibitor); however, we found a different behavior with *Fh*CL3. Here, the progress curves have a lag phase followed by a linear phase (Fig 2, right panel), reflecting the slow recovery of activity as inhibitor dissociates from the enzyme and suggesting that the interaction of **C34** with *Fh*CL3 is slowly reversible being the compound tightly bound to the enzyme.

### Flavonoids can bind along the active site

**Fig 3:**
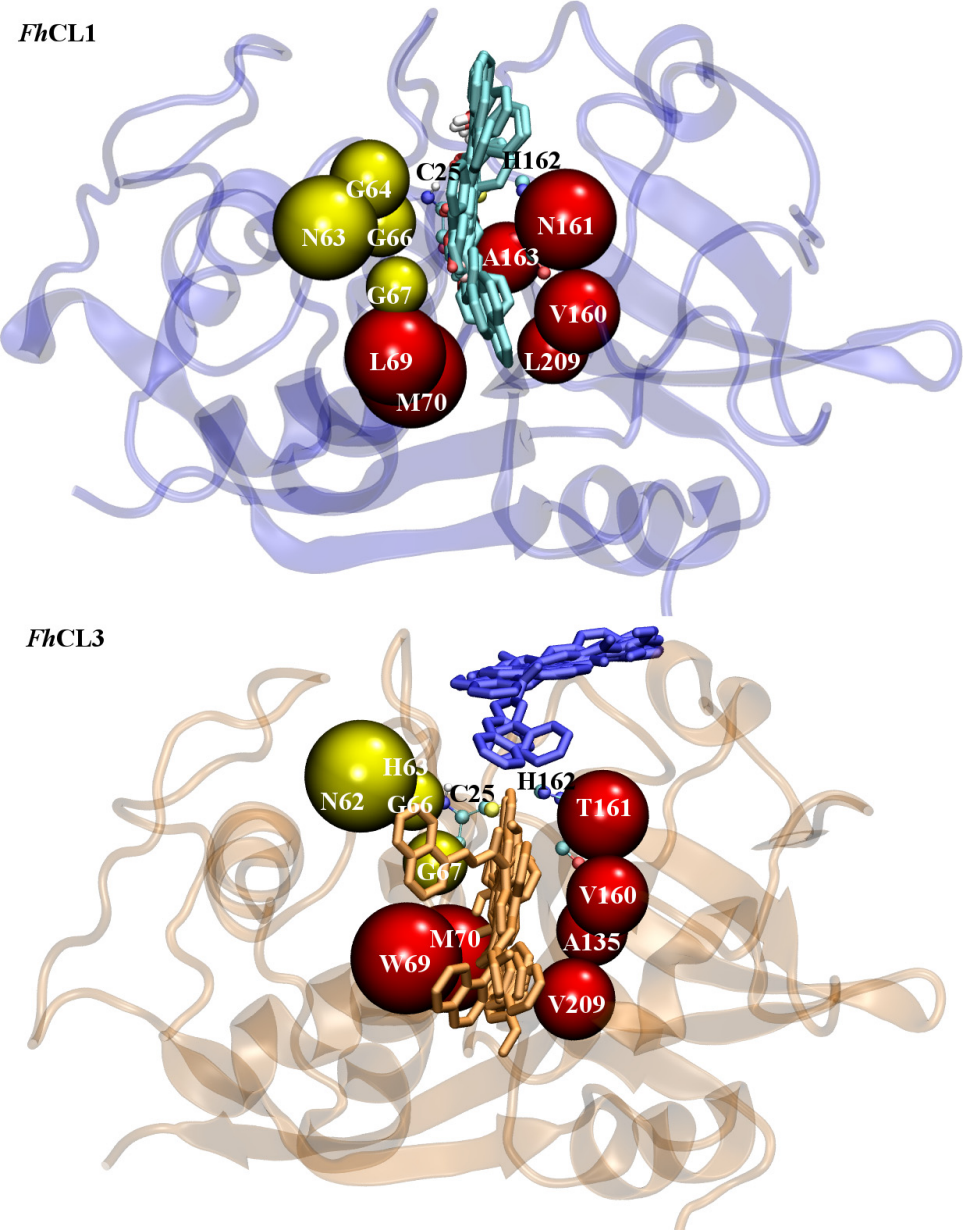
Flavonoids binding sites predicted by molecular docking. Flavonoid interaction is predicted to occur along the active site cleft of the target enzymes. **Above**: Site represented with cyan compounds superimposed has 100% occupation with flavonoids evaluated in *Fh*CL1. **Below:** Sites represented in orange and blue are the more populated clusters found in *Fh*CL3. Around 40% of compounds have only one cluster (represented in orange) while about 33% of flavonoids have also a second cluster with similar probability (colored in blue). **C34** is included in the second group. Enzyme structures are represented by new cartoon and compounds structures in licorice. The catalytic dyad Cys^25^ and His^162^ is represented in balls and sticks. Subsites residues are depicted in beads colored in red (S_2_) and yellow (S_3_).

According to docking results the studied compounds most frequently interact with a putative binding site, represented in cyan for *Fh*CL1 and orange for *Fh*CL3, and found at a similar position within the active site cleft of each enzyme (Fig 3). A second putative binding site (represented in blue) was observed for *Fh*CL3, which has a different orientation and is not as deep located inside the binding site (Fig 3).

**Fig 4:**
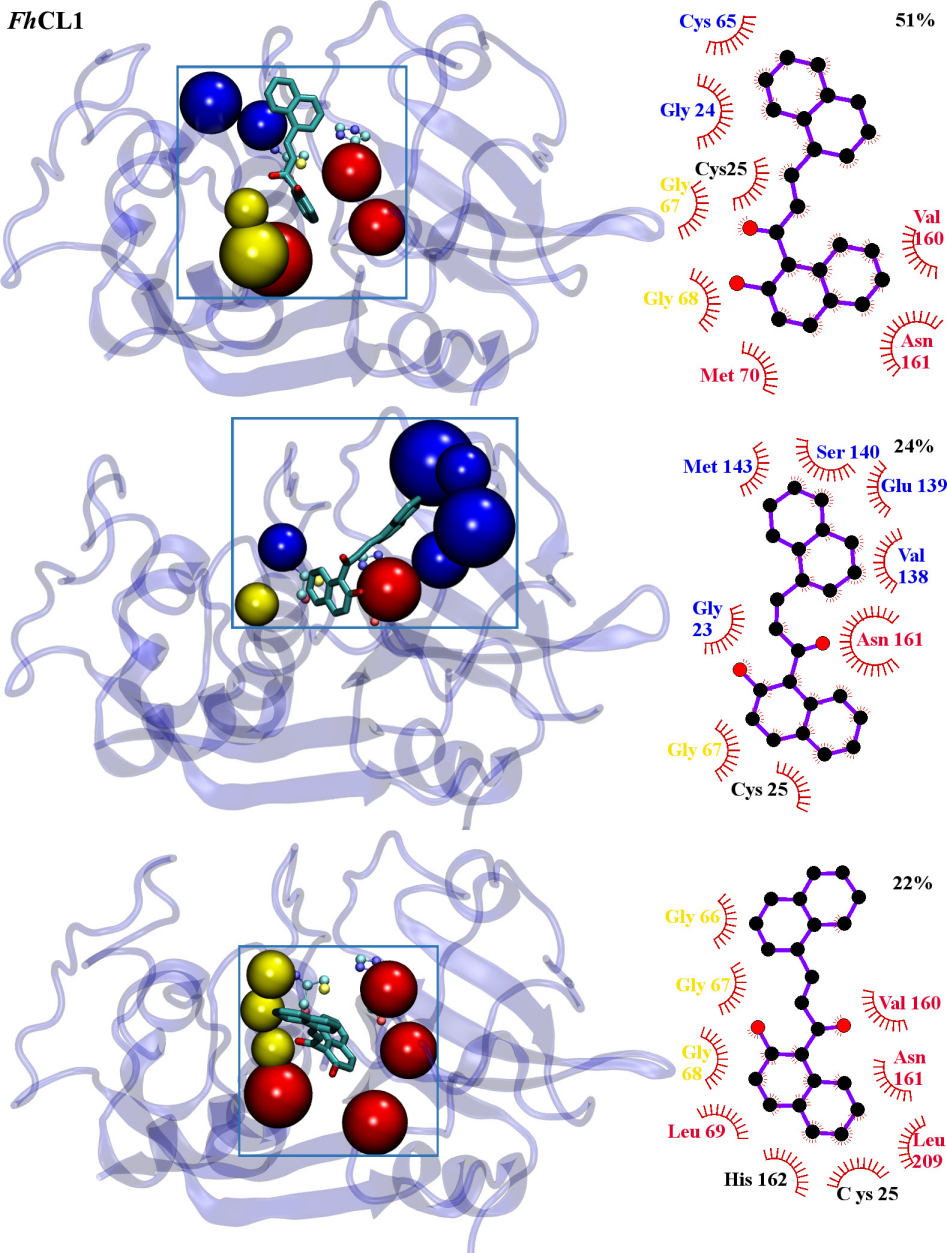
*Fh*CL1-C34 interactions from MD simulations. Structure of the three most populated clusters for *Fh*CL1. **Left panels: C34** structure is represented in balls and sticks. Residues belonging to sub-sites involved on complex formation are represented in beads and colored in red (S_2_) and yellow (S_3_). Residues outside the active site pockets involved on complex formation are translucent (light blue). Catalytic dyad is depicted by VDW. **Right panels:** 2D diagram of interactions between **C34** and *Fh*CL1 for each cluster generated using LigPlot+ [60]. **C34** is shown in violet. Protein residues involved in non-polar contacts are represented as red semicircles with radiating spokes and their names colored according to left panels. A hydrogen bond (green dashed line) is shown together with its distance value (green).

**Fig 5:**
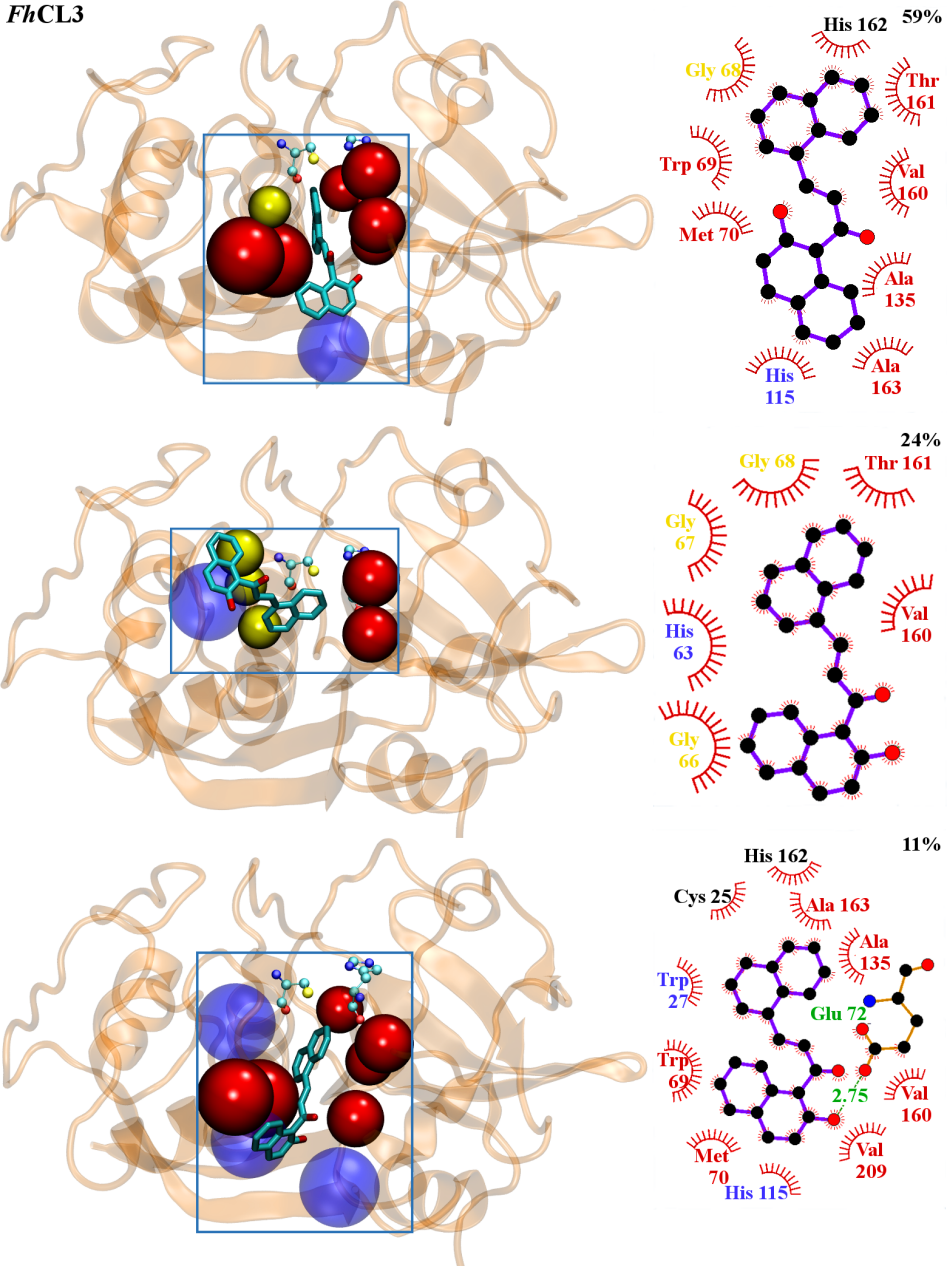
*Fh*CL3-C34 interactions from MD simulations. Structure of the three most populated clusters for *Fh*CL3. **Left panels: C34** structure is represented in balls and sticks. Residues belonging to sub-sites involved on complex formation are represented in beads and colored in red (S_2_) and yellow (S_3_). Residues outside the active site pockets involved on complex formation are translucent (light blue). Catalytic dyad is depicted by VDW. **Right panels:** 2D diagram of interactions between **C34** and *Fh*CL3 for each cluster generated using LigPlot+. **C34** is shown in violet. Protein residues involved in non-polar contacts are represented as red semicircles with radiating spokes and their names colored according to left panels. A hydrogen bond (green dashed line) is shown together with its distance value (green).

To obtain a deeper knowledge of **C34** interaction with target enzymes, 40 ns molecular dynamics simulations were performed allowing us to confirm the binding sites predicted by docking, as we obtained stable complexes in the last 35 ns of the simulation (S1 Fig). Thus, the high percentage of inhibition experimentally observed can be attributed to the compound locating next to residues directly implicated in substrate positioning and catalysis (Figs 4 and 5). From a population cluster analysis, we determined the most representative structure for the enzyme-inhibitor complex for *Fh*CL1, with an occurrence of 51% (Fig 4 upper panel). We found two more clusters with occurrences of 24% and 22% that, while changing the relative position and interaction with certain residues, still occupy the active site (Fig 4 middle and lower panels, respectively), and two last clusters with lower occurrences of 2 and 1% (not shown). For *Fh*CL3, **C34** occupies the orange site (Fig 3) along the entire simulation time. A major cluster with an occurrence of 59% is observed (Fig 5 upper panel), followed by another two clusters with lower occurrences of 23% and 11% (Fig 5 middle and lower panels, respectively), and two with less occurrences of 6 and 1% (not shown), similar in location and relative position to the major cluster.

The experimentally observed variation in inhibition percentages might be explained by the high number of interactions established by the ligand with each target. It is worth noticing that the experimental results were in good correlation with computational observations. **C34** is positioned along the active site cleft in both enzymes and interacts with Cys-His catalytic dyad and residues from the S_2_ and S_3_ pockets (Figs 4 and 5), determinants of the substrate specificity of this family of cysteine proteases [39, 61]. *Fh*CL1 establishes several hydrophobic interactions with the inhibitor, particularly through residues Gly^66^, Gly^67^, Val^160^ and Asn^161^ from S_2_ and S_3_ subsites, and Cys^25^ from the catalytic dyad (Fig 4). Likewise, we observed many hydrophobic interactions of **C34** with *Fh*CL3, with a larger number of residues belonging to the enzyme sub-sites: Trp^69^, Met^70^, Ala^135^, Val^160^, Thr^161^, Val^163^ and Ala^209^, which form the entire S_2_ subsite, plus Gly^68^ belonging to S_3_ subsite, both decisive in *Fh*CL3 substrate positioning for catalysis (Fig 5). In this sense, the high inhibition experimentally observed for **C34** with both enzymes is confirmed.

Interestingly, Trp^69^ can contribute to inhibitor binding by establishing Pi-Pi stacking interactions. This residue is specific of *Fh*CL3 and when mutated for Leu as in *Fh*CL1 it renders the enzyme almost inactive highlighting its importance for substrate positioning for catalysis [39]. A recent *in silico* search for *Fh*CL3 inhibitors provides additional evidence that the non-polar side chain of Trp^69^ establishes critical interaction with ligands and adopts variable conformations to accommodate different groups in the enzyme binding site [62]. Furthermore, it showed that aromatic moieties with high hydrophobicity can establish favorable interactions with non-polar residues from *Fh*CL3 binding cleft [62]. The greater number of interactions between inhibitor and residues from *Fh*CL3 sub-sites compared with *Fh*CL1, might explain its lower IC_50_ (Table 1), its behavior as a slowly reversible inhibitor (Fig 2), and its highest percentage of inhibition reached after 120 min (Table 1).

**Fig 6:**
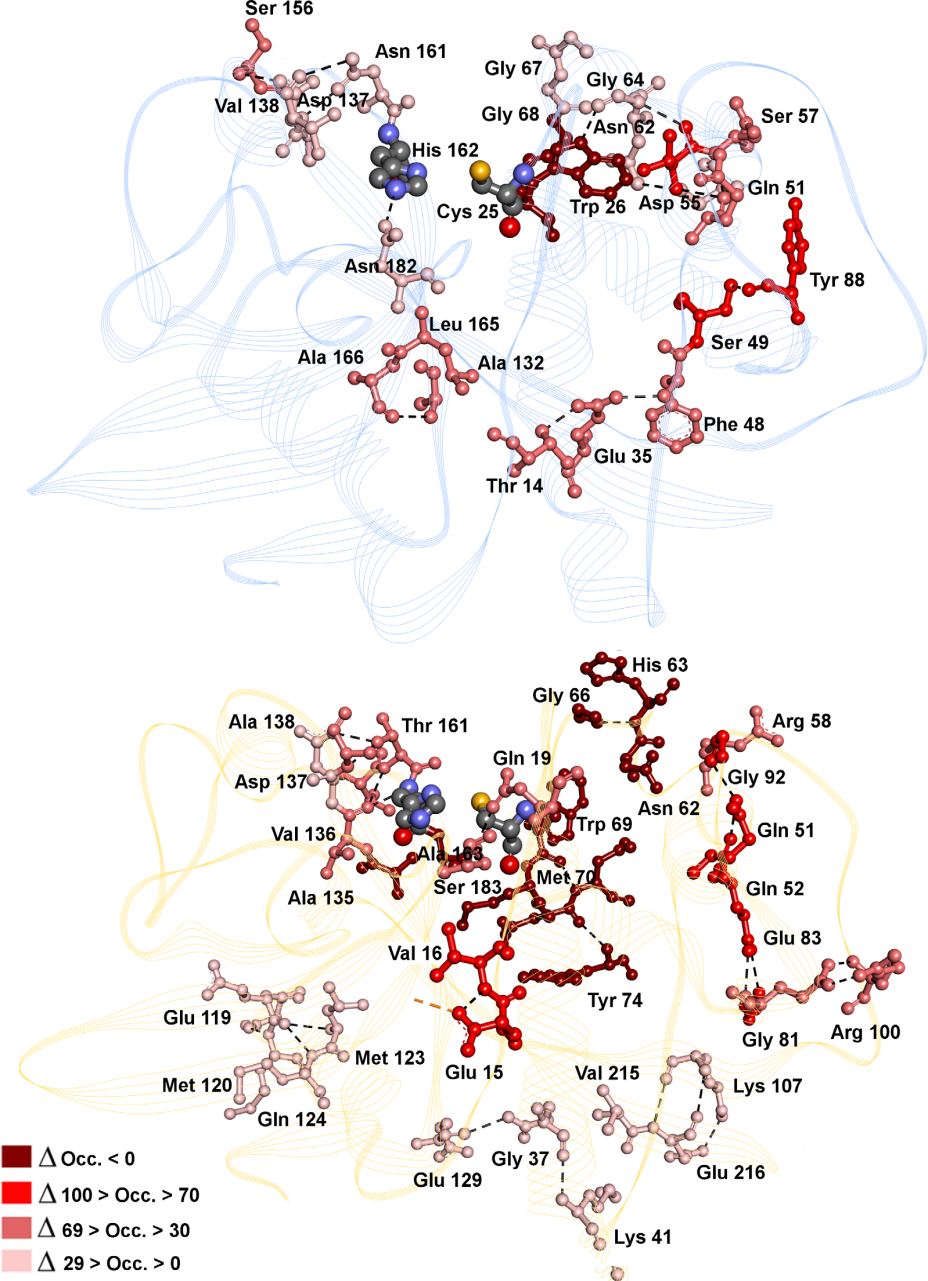
Hydrogen bonds varying in the presence or absence of C34 bound to *Fh*CL1 (blue, top panel) and *Fh*CL3 (orange, low panel). Residues involved in hydrogen bonds (HB) are depicted in ball and sticks and colored by ΔOcc (delta of occupancy). ΔOcc is calculated with the formula ΔOcc = Occ *Fh*CL—Occ *Fh*CL_34 from the data in S5 and S6 Tables. HB are represented by black dashed lines. The catalytic Cys-His dyad is colored by atom.

No hydrogen bonds with a significant occupancy (greater than 50) along the simulation time were found in any of the enzyme-inhibitor complexes, except for the bond between carbonyl oxygen in **C34** and Gly^68^ with an occupancy of 22 in *Fh*CL1 (Fig 4), analogous to the interaction with Glu^72^ in *Fh*CL3, with only 11 of occupancy (Fig 5). However, there are significant differences in the hydrogen bonds at the protein structural level, where some intra-molecular protein hydrogen bonds have an occupancy greater than 90 in the absence of inhibitor but disappear in its presence, mainly in the *Fh*CL3-inhibitor complex (Fig 6).

If we analyze the hydrogen bonds involving residues from sub-sites S_2_ and S_3_ in *Fh*CL1 (Fig 6 and S5 Table, residues in bold), we see a tendency to diminish its occupancy in the presence of **C34**, supporting its interaction along the active site alters local protein conformation. A similar behavior is observed in *Fh*CL3, with a more remarkable loss in occupancy seen for hydrogen bonds involving Val^136^-Thr^161^, Asp^137^- Thr^161^ and His^63^-Arg^58^ (Fig 6 and S6 Table, residues in bold). Moreover, upon ligand binding new hydrogen bonds are established between backbone and side chains of sub-site residues (with occupancy greater than 50) in the proximity of **C34** binding site (Fig 6, HB with ΔOcc.˂0), named His^63^-Gly^66^, Trp^69^-Asn^62^ and Ala^135^-Ala^163^ (S6 Table, residues in bold), again suggesting the interaction with **C34** dramatically alters protein conformation and thus its ability for catalysis.

### Fasciolicide activity of C34 over *in vitro* cultured NEJ

**Fig 7:**
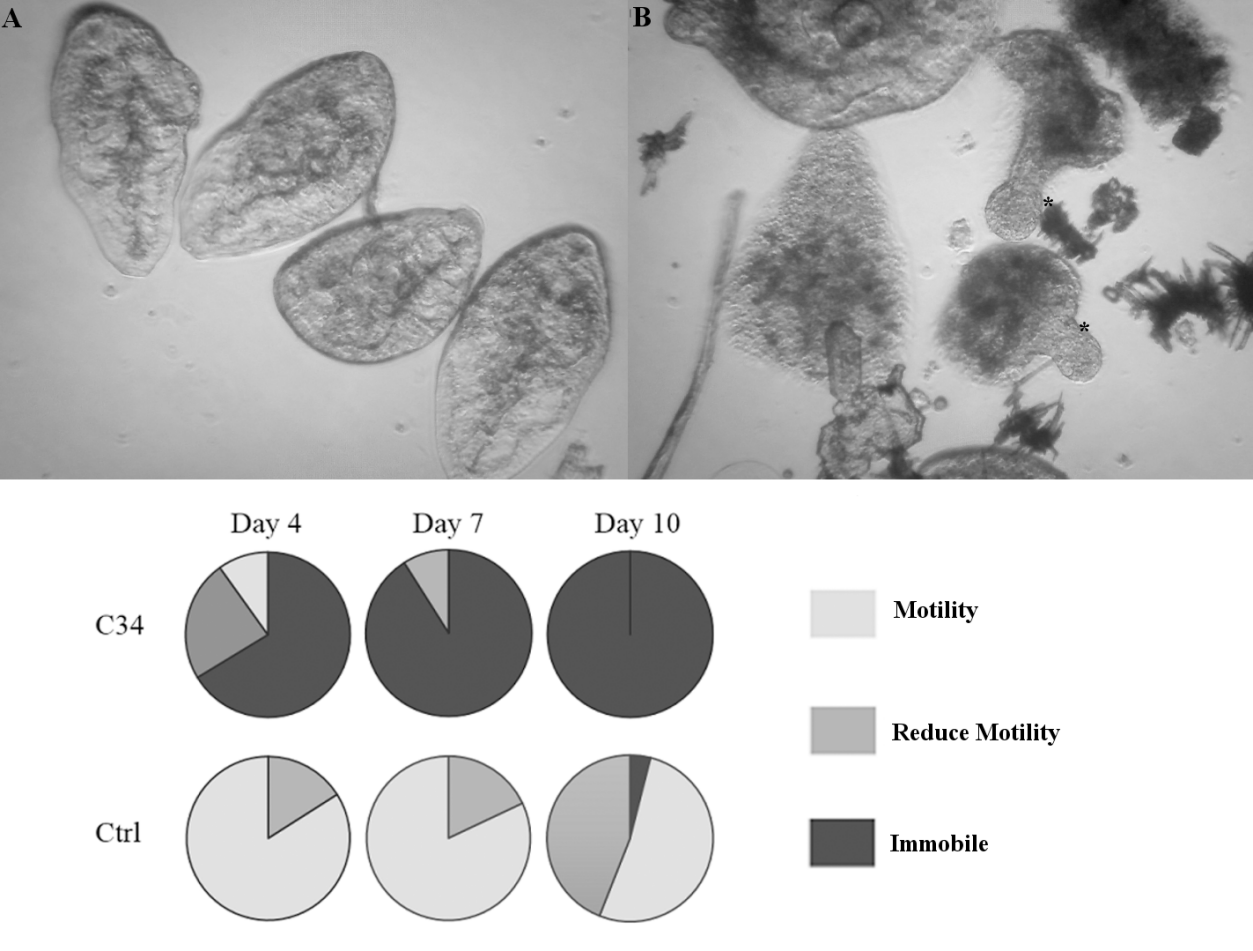
Viability assessment of NEJ cultured with C34. Top: Microscopic appearance of control (left) and treated parasites (right) after 7 days of culture. * NEJ dead with protrusions. Bottom: pie charts showing the percentage of NEJ in each score category when incubated with 50 μM **C34** (duplicates taken together for percentage calculation): 3-normally active; 2- reduced activity (sporadic movement); 1- immobile. Control NEJ were incubated in 0.5% DMSO.

We evaluated the effect of **C34** on NEJ parasites cultured *in vitro*. NEJ were incubated in the presence of 50 μM **C34** and the assessment of parasite movement is shown in Fig 7.

The addition of **C34** resulted in a decrease in parasite motility leading to parasite death. At day 4 the percentage of immobile flukes was considerably higher in treated *vs* control groups, 67% *vs* 0% respectively. The percentage of sporadic movement and immobile parasites increased in treated parasites and evident signs of internal and tegument damage appeared. Culture media was dirty as compared to control flukes due to the release of body components and some NEJ died protruding their anterior region (Fig 7, S1 and S2 Videos). After day 10 no movement was detected in treated NEJ while control parasites continue moving until the experiment was finished on day 20, thus, motility seems to adequately estimate worm viability.

**Fig 8:**
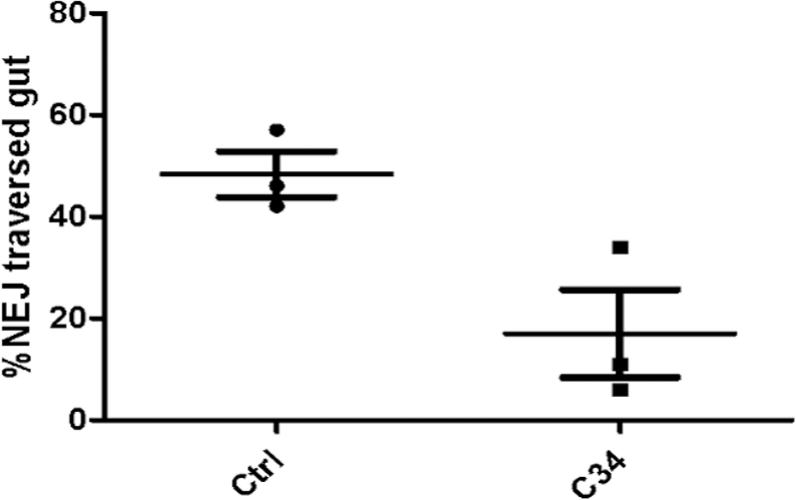
NEJ gut penetration assay. Scattered plot showing the percentage of NEJ that traverse the gut wall within 3 h in control (circles) and **C34** treated groups (squares). Horizontal lines and bars represent means ± SD percent gut penetration, respectively.

There have been controversial findings regarding the effect of cathepsin L *knock down* by RNAi. While a recent work described no phenotypic changes in NEJ even after 21 days of exposure to long dsRNA or siRNA [63], an earlier report had documented immobile NEJ after a 4h interference with Cat-L and B siRNA [64]. Here, we observed a reduction in fluke motility consistent with this first report. Additional evidence supporting this result comes from an *in vitro* treatment of NEJ with the cathepsin L and B inhibitors E64-d and CA-074Me, which resulted in the loss of worm motility accompanied by structural damage of parasites [65]. In a similar fashion, they observed a progressive loss of motility starting 5 to 12 days after treatment initiation. As a consequence of decreased motility, a diminished ability of parasites to traverse the duodenum wall was reported [64]. In order to check if **C34** treatment can also impair invasion, we performed a gut penetration assay. NEJ incubated 4h with **C34** showed the expected phenotype of reduced migration through the gut wall, suggesting the impairment of cathepsin L function (Fig 8). While the *in vitro* treatment effect of movement reduction is seen after longer incubation times with **C34**, a reduced gut penetration is readily detected in the invasion assay. This is indicative that the main role of juvenile cathepsin L3 in parasite host invasion is promptly affected, and a secondary effect on parasite movement might emerge later. In the host environment, this might translate into a considerable reduction in the number of NEJ able to reach the bile ducts and establish a successful infection.

### C34 is not toxic to bovine spermatozoa

Citotoxicity assays using spermatozoa are considered a suitable approach for preclinical toxicology screening during drug development processes [66]. The cytotoxicity assay on bovine spermatozoa showed **C34** is not toxic to reproductive cells even at the highest tested dose of 100 μM. We observed no significant differences on percentage of motile spermatozoa and velocity curved line between treated and control samples (S7 Table). Also, our previous studies showed **C34** is not mutagenic in Ames test using *Salmonella typhimurium* TA98 strain with and without metabolic activation and no genotoxic effect was observed measuring DNA damage in the alkaline comet assay [36], supporting the fact that this class of compounds exhibits low toxicity and are promising agents for clinical usage.

### Conclusions

In this work we have identified novel chalcones that inhibit *Fasciola hepatica* major cathepsins L from both adult and juvenile parasites. Chalcones with heterocycles as B-ring (**C22-C26**) and chalcones containing phenyl and naphtyl moieties (**C27**-**C35**) showed the highest inhibition percentages. Among these, **C34** resulted a slow-reversible tight binding inhibitor of *Fh*CL3, exerting an almost complete inactivation of the enzyme. In addition, we performed an extensively computational analysis characterizing **C34** interaction with cathepsins providing evidence of the different inhibition seen with each target enzyme. We demonstrated **C34** is not toxic to bovine sperm, exhibits *in vitro* fasciolicide activity over cultured NEJ and reduces larval penetration of the gut wall, suggesting is a suitable candidate for further drug development against worm infection.

## Supporting Information

S1 Fig Root mean squared deviations (RMSDs) of backbone atoms of *Fh*CL1 and *Fh*CL3 (black line) and enzyme complexes with C34 (grey line). (TIF)

S1 Table Structure of chalcones modified on ring B (cluster I) and its inhibition percentages in *Fh*CL1 and *Fh*CL3. % inh.: percentage of inhibition at 10 μM dose. Values represent means ± SE. *n* = 2. R: represent B ring.
(DOCX)

S2 Table Structure of naphtochalcone derivatives (cluster II) and its inhibition percentages in FhCL1 and FhCL3. % inh.: percentage of inhibition at 10 μM dose. Values represent means ± SE. *n* = 2. *C29 is a derivative of C28, which lacks the α,β-unsaturated system: 3-(naphthalen-1-yl)-1,5-diphenylpentane-1,5-dione.
(DOCX)

S3 Table Structure of flavone derivatives substituted on ring B (cluster III) and its inhibition percentages in *Fh*CL1 and *Fh*CL3. % inh.: percentage of inhibition at 10 μM dose. Values represent means ± SE. *n* = 2.
(DOCX)

S4 Table Comparison of inhibition percentage and hydrogen bond (HB) prediction between enzymes and C22-C25. (DOCX)

S5 Table Hydrogen bonds of *Fh*CL1 varying in presence or absence of C34. Residues of catalytic sub-sites are highlighted in bold. ΔOcc is calculated with the formula ΔOcc = Occ *Fh*CL1—Occ *Fh*CL1_34
(DOCX)

S6 Table Hydrogen bonds of *Fh*CL3 varying in presence or absence of C34. Residues of catalytic sub-sites are highlighted in bold. ΔOcc is calculated with the formula ΔOcc = Occ *Fh*CL3—Occ *Fh*CL3_34.
(DOCX)

S7 Table Cytotoxicity assay on bovine spermatozoa. % MOT: percentage of motile spermatozoa (motility > 5 μm/s) normalized to untreated control. VCL: Velocity curved line (> 24 μm/s). Spermatozoa were treated 1h with **C34** at 37°C.
(DOCX)

S1 Video Control NEJ cultured for 10 days recorded with DINO-EYE Microscope Eye-Piece Camera AM-4023X. (WMV)

S2 Video NEJ cultured 7 days with 50 μM C34 recorded with DINO-EYE Microscope Eye-Piece Camera AM-4023X. (WMV)
