# Supplementary figures and images for: Identification of Chalcones as Fasciola hepatica Cathepsin L Inhibitors Using a Comprehensive Experimental and Computational Approach
Source: PLoS Negl Trop Dis. 2016 Jul 27;10(7):e0004834. doi: 10.1371/journal.pntd.0004834 (PMC4962987; doi:10.1371/journal.pntd.0004834)

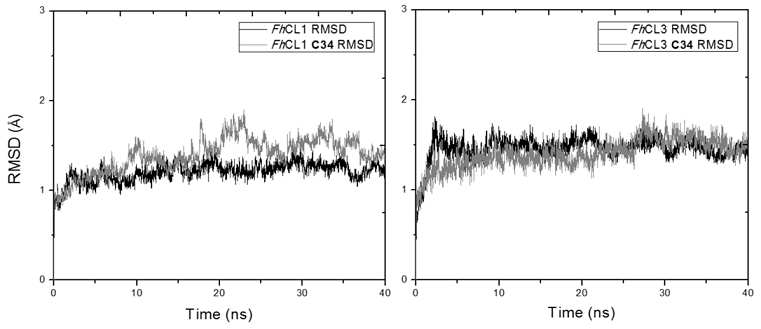

Supplement: S1 Fig — (TIF) [file pntd.0004834.s001.tif]
