# Supplementary material for: Identification of Chalcones as Fasciola hepatica Cathepsin L Inhibitors Using a Comprehensive Experimental and Computational Approach
Source: PLoS Negl Trop Dis. 2016 Jul 27;10(7):e0004834. doi: 10.1371/journal.pntd.0004834 (PMC4962987; doi:10.1371/journal.pntd.0004834)
Supplement: S1 Table — % inh.: percentage of inhibition at 10 μM dose. Values represent means ± SE. n = 2. R: represent B ring. (DOCX) [file pntd.0004834.s002.docx]

|  | | | | |
| --- | --- | --- | --- | --- |
|  | | | | |
| **Cpd.** | **X** | **R** | **% inh. *Fh*CL1** | **% inh. *Fh*CL3** |
| **C1** | H | -Ph | 33 ± 8 | 4 ± 4 |
| **C2** | H | -Ph-4-Cl | 25 ± 12 | 6 ± 5 |
| **C3** | H | -Ph-4-Br | 52 ± 5 | 12 ± 14 |
| **C4** | H | -Ph-4-OCH_3_ | 48 ± 8 | 4 ± 5 |
| **C5** | H | -Ph-3,4-di-OCH_3_ | 35 ± 5 | 2 ± 7 |
| **C6** | H | -Ph-4-SCH_3_ | 8 ± 9 | 26 ± 1 |
| **C7** | H | -Ph-4-NHCOCH_3_ | 14 ± 34 | 12 ± 6 |
| **C8** | H | -Ph-4-NH_2_.HCl | 4 ± 19 | 2 ± 6 |
| **C9** | OH | -Ph | 16 ± 6 | 0 ± 8 |
| **C10** | OH | -Ph-4-Cl | 63 ± 10 | 23 ± 3 |
| **C11** | OH | -Ph-4-Br | 19 ± 2 | 12 ± 7 |
| **C12** | OH | -Ph-2-Br | 29 ± 3 | 33 ± 7 |
| **C13** | OH | -Ph-4-OCH_3_ | 48 ± 9 | 3 ± 5 |
| **C14** | OH | -Ph-3-I,4-OCH_3_ | 0 ± 9 | 19 ± 1 |
| **C15** | OH | -Ph-4-SCH_3_ | 6 ± 11 | 32 ± 1 |
| **C16** | OH | -Ph-4-NHCOCH_3_ | 0 ± 10 | 13 ± 1 |
| **C17** | OH | -Ph-4-N(CH_3_)_2_ | 34 ± 12 | 20 ± 4 |
| **C18** | OH | -Ph-4-OBn | 8 ± 13 | 16 ± 1 |
| **C19** | OH | -Ph-3-NO_2_ | 26 ± 6 | 37 ± 1 |
| **C20** | H | -2-pyridinyl | 8 ±16 | 4 ± 1 |
| **C21** | H | -5-benzofuroxanyl | 21 ± 9 | 26 ± 1 |
| **C22** | OH | -2-pyridinyl | 54 ± 7 | 0 ± 10 |
| **C23** | OH | -2-furyl | 57 ± 8 | 4 ± 10 |
| **C24** | OH | -2-thienyl | 52 ± 5 | 9 ± 11 |
| **C25** | OH | -4-furoxanyl | 48 ± 6 | 14 ± 12 |
| **C26** | OH | -5-benzodioxolyl | 11 ± 14 | 19 ± 6 |
|  | | | | |
