# Supplementary material for: Identification of Chalcones as Fasciola hepatica Cathepsin L Inhibitors Using a Comprehensive Experimental and Computational Approach
Source: PLoS Negl Trop Dis. 2016 Jul 27;10(7):e0004834. doi: 10.1371/journal.pntd.0004834 (PMC4962987; doi:10.1371/journal.pntd.0004834)
Supplement: S2 Table — % inh.: percentage of inhibition at 10 μM dose. Values represent means ± SE. n = 2. *C29 is a derivative of C28, which lacks the α,β-unsaturated system: 3-(naphthalen-1-yl)-1,5-diphenylpentane-1,5-dione. (DOCX) [file pntd.0004834.s003.docx]

|  | | | | | | |
| --- | --- | --- | --- | --- | --- | --- |
|  | | | | | | |
| **Cpd.** | **X** | **A ring** | **-C=O position** | **B ring** | **% inh. *Fh*CL1** | **% inh. *Fh*CL3** |
| **C27** | H | naphthyl | 2 | phenyl | 65 ± 3 | 13 ± 4 |
| **C28** | H | phenyl | - | 1-naphthyl | 38 ± 3 | 39 ± 5 |
| **C29** | H | phenyl | * | 1-naphthyl | 17 ± 9 | 27 ± 2 |
| **C30** | H | phenyl | - | 2-naphthyl | 62 ± 2 | 38 ± 7 |
| **C31** | OH | phenyl | - | 2-naphthyl | 61 ± 2 | 41 ± 5 |
| **C32** | OH | phenyl | - | 1-naphthyl | 42 ± 6 | 32 ± 8 |
| **C33** | 1-OH | naphthyl | 2 | 2-naphthyl | 65 ± 6 | 29 ± 5 |
| **C34** | 2-OH | naphthyl | 1 | 1-naphthyl | 75 ± 4 | 65 ± 6 |
| **C35** | 2-OH | naphthyl | 1 | 2-naphthyl | 67 ± 3 | 44 ± 6 |
|  | | | | | | |
