# Supplementary material for: Identification of Chalcones as Fasciola hepatica Cathepsin L Inhibitors Using a Comprehensive Experimental and Computational Approach
Source: PLoS Negl Trop Dis. 2016 Jul 27;10(7):e0004834. doi: 10.1371/journal.pntd.0004834 (PMC4962987; doi:10.1371/journal.pntd.0004834)
Supplement: S3 Table — % inh.: percentage of inhibition at 10 μM dose. Values represent means ± SE. n = 2. (DOCX) [file pntd.0004834.s004.docx]

|  | | | |
| --- | --- | --- | --- |
|  | | | |
| **Cpd.** | **R** | **% inh. *Fh*CL1** | **% inh. *Fh*CL3** |
| **C36** | -H | 5 ± 1 | 8 ± 4 |
| **C37** | -Cl | 0 ± 11 | 17 ± 1 |
| **C38** | -Br | 21 ± 10 | 19 ± 3 |
| **C39** | -OCH_3_ | 20 ± 7 | 0 ± 3 |
| **C40** | Quercetin | 15 ± 8 | 8 ± 1 |
|  | | | |
