# Supplementary material for: Identification of Chalcones as Fasciola hepatica Cathepsin L Inhibitors Using a Comprehensive Experimental and Computational Approach
Source: PLoS Negl Trop Dis. 2016 Jul 27;10(7):e0004834. doi: 10.1371/journal.pntd.0004834 (PMC4962987; doi:10.1371/journal.pntd.0004834)
Supplement: S4 Table — (DOCX) [file pntd.0004834.s005.docx]

| **Cpd.** | **% inh. *Fh*CL1** | **% inh. *Fh*CL3** | **HB *Fh*CL1** | **HB *Fh*CL3** |
| --- | --- | --- | --- | --- |
| **C22** | 54 ± 7 | 0 ± 10 | Ala 163/ Trp 26 | No |
| **C23** | 57 ± 8 | 4 ± 10 | Trp 27 | No |
| **C24** | 52 ± 5 | 9 ± 11 | No | No |
| **C25** | 48 ± 6 | 14 ± 12 | **Gly 68** | Glu 71 |
