# Supplementary material for: Identification of Chalcones as Fasciola hepatica Cathepsin L Inhibitors Using a Comprehensive Experimental and Computational Approach
Source: PLoS Negl Trop Dis. 2016 Jul 27;10(7):e0004834. doi: 10.1371/journal.pntd.0004834 (PMC4962987; doi:10.1371/journal.pntd.0004834)
Supplement: S5 Table — Residues of catalytic sub-sites are highlighted in bold. ΔOcc is calculated with the formula ΔOcc = Occ FhCL1—Occ FhCL1_34 (DOCX) [file pntd.0004834.s006.docx]

| Acceptor | Donor H | Donor | Occ. *Fh*CL1 | Occ. *Fh*CL1_34 | ΔOcc. |
| --- | --- | --- | --- | --- | --- |
| GLU_35:OE1 | THR_14:HG1 | THR_14:OG1 | 99 | 49 | 50 |
| ASP_137:OD2 | SER_156:H | SER_156:N | 99 | 36 | 63 |
| TYR_88:O | SER_49:HG | SER_49:OG | 93 | 17 | 76 |
| GLU_35:OE2 | PHE_48:H | PHE_48:N | 93 | 42 | 51 |
| GLN_51:O | ASP_55:H | ASP_55:N | 93 | 15 | 78 |
| ASN_182:OD1 | **HIS_162:HE2** | **HIS_162:NE2** | **75** | **53** | **22** |
| ALA_166:O | ALA_132:H | ALA_132:N | 69 | 35 | 34 |
| SER_57:OG | **ASN_62:HD22** | **ASN_62:ND2** | **67** | **40** | **27** |
| VAL_138:O | **ASN_161:HD21** | **ASN_161:ND2** | **54** | **36** | **18** |
| GLY_64:O | **GLY_67:H** | **GLY_67:N** | **51** | **32** | **19** |
| GLY_64:O | TRP_26:HE1 | TRP_26:NE1 | 44 | 72 | -28 |
| ASP_137:OD1 | **ASN_161:H** | **ASN_161:N** | **44** | **23** | **21** |
| **34**_220:O1 | **GLY_68:H** | **GLY_68:N** | - | **22** | **-22** |
