# Supplementary material for: Identification of Chalcones as Fasciola hepatica Cathepsin L Inhibitors Using a Comprehensive Experimental and Computational Approach
Source: PLoS Negl Trop Dis. 2016 Jul 27;10(7):e0004834. doi: 10.1371/journal.pntd.0004834 (PMC4962987; doi:10.1371/journal.pntd.0004834)
Supplement: S6 Table — Residues of catalytic sub-sites are highlighted in bold. ΔOcc is calculated with the formula ΔOcc = Occ FhCL3—Occ FhCL3_34. (DOCX) [file pntd.0004834.s007.docx]

| Acceptor | Donor H | Donor | Occ. *Fh*CL3 | Occ. *Fh*CL3_34 | ΔOcc. |
| --- | --- | --- | --- | --- | --- |
| GLN_19:OE1 | SER_183:HG | SER_183:OG | 99 | 46 | 53 |
| GLN_52 :OE1 | GLU_83:H | GLU_83:N | 97 | 85 | 12 |
| GLU_129:O | GLN_37:HE21 | GLN_37:NE2 | 96 | 83 | 13 |
| GLU_15:OE1 | VAL_16:H | VAL_16:N | 96 | 1 | 95 |
| GLY_92:O | GLN_51:HE21 | GLN_51:NE2 | 92 | 0 | 92 |
| GLU_119:O | MET_123:H | MET_123:N | 92 | 73 | 19 |
| GLU_83:OE1 | ARG_100:HH21 | ARG_100:NH2 | 91 | 56 | 35 |
| MET_120:O | GLN_124:H | GLN_124:N | 90 | 82 | 8 |
| SER_183:O | GLN_19:H | GLN_19:N | 90 | 36 | 54 |
| GLU_216:O | LYS_107:H | LYS_107:N | 90 | 88 | 2 |
| GLN_37:O | LYS_41:H | LYS_41:N | 89 | 64 | 25 |
| GLY_81:O | GLN_52:HE21 | GLN_52:NE2 | 81 | 1 | 80 |
| VAL_136:O | **THR_161:H** | THR_161:N | 77 | 39 | **38** |
| **THR_161:OG1** | ALA_138:H | ALA_138:N | 71 | 28 | **43** |
| **HIS_63:O** | ARG_58:HH11 | ARG_58:NH1 | 46 | 6 | **40** |
| ASP_137:OD1 | **THR_161:HG1** | **THR_161:OG1** | **45** | **21** | **24** |
| ALA_135:HA | **ALA_163:HA** | **ALA_163:CA** | **24** | **54** | **-30** |
| **MET_70:O** | TYR_74:H | TYR_74:N | **2** | **40** | **-38** |
| **HIS_63:ND1** | **GLY_66:H** | **GLY_66:N** | **1** | **59** | **-58** |
| **ASN_62:OD1** | **TRP_69:H** | **TRP_69:N** | **1** | **46** | **-45** |
| **TRP_69:HZ3** | **ASN_62:H** | **ASN_62:N** | **1** | **58** | **-57** |
| **ALA_135:HB2** | **ALA_163:HA** | **ALA_163:CA** | **0** | **54** | **-54** |
| GLU_72:OE1 | **34**_220:H16 | **34**_220:O2 | - | 11 | **-11** |
