# Supplementary material for: Identification of Chalcones as Fasciola hepatica Cathepsin L Inhibitors Using a Comprehensive Experimental and Computational Approach
Source: PLoS Negl Trop Dis. 2016 Jul 27;10(7):e0004834. doi: 10.1371/journal.pntd.0004834 (PMC4962987; doi:10.1371/journal.pntd.0004834)
Supplement: S7 Table — % MOT: percentage of motile spermatozoa (motility > 5 μm/s) normalized to untreated control. VCL: Velocity curved line (> 24 μm/s). Spermatozoa were treated 1h with C34 at 37°C. (DOCX) [file pntd.0004834.s008.docx]

| **C34 (µM)** | **% MOT** | **VCL (µm/s)** |
| --- | --- | --- |
| 0 | 100 ± 14 | 63 ± 6 |
| 6.25 | ˃ 90 | ˃ 60 |
| 12.5 | ˃ 90 | ˃ 60 |
| 25 | ˃ 90 | ˃ 60 |
| 50 | ˃ 90 | ˃ 60 |
| 100 | ˃ 90 | ˃ 60 |
